# A Novel Zn-Cu Bimetallic Mixed-Component MOFs Composite for Efficient CO_2_ Capture

**DOI:** 10.3390/nano15231777

**Published:** 2025-11-26

**Authors:** Haihong Zhao, Lei Li, Jiaxin Li, Feiqi Yan, Wenhao Wang, Mingxia Zhao

**Affiliations:** 1 Department of Mining Engineering, Shanxi Institute of Technology, Yangquan 045000, China; 2 Yangquan Technology Innovation Center of Carbon Dioxide Capture, Utilization and Storage, Yangquan 045000, China; 3 State Key Laboratory of Coal Conversion, Institute of Coal Chemistry, Chinese Academy of Sciences, Taiyuan 030001, China

**Keywords:** metal–organic framework, Zn-Cu-BTC/MCFs, CO_2_ capture, adsorption selectivity

## Abstract

In this work, a novel mixed-component bimetallic metal–organic framework (MOF) composite material was synthesized via a solvothermal approach, and its structural and textural properties were systematically characterized using X-ray diffraction (XRD), scanning electron microscopy (SEM), N_2_ adsorption/desorption analysis, and transmission electron microscopy (TEM). Furthermore, the single-component adsorption isotherms of CO_2_ and N_2_ were experimentally measured and fitted to the Langmuir–Freundlich model. The CO_2_/N_2_ selectivity of the composite was evaluated based on the ideal adsorption solution theory (IAST). The results demonstrated that the addition of Zn^2+^ significantly enhanced the specific surface area and improved the CO_2_ adsorption capacity (3.97 mmol/g at 35 °C and 1 bar), with an increase of 31.5% in comparison with the Cu-BTC/MCFs (3.02 mmol/g). Meanwhile, the Zn-Cu-BTC/MCFs had good recyclability and CO_2_/N_2_ selectivity up to 12.5 determined via IAST (CO_2_:N_2_ = 85:15).

## 1. Introduction

The excessive emission of carbon dioxide resulting from human activities is widely recognized as the primary contributor to the global greenhouse effect [1]. Therefore, the exploitation of CO_2_ reduction technologies is crucial from the perspective of sustainable development. Currently, CO_2_ capture technologies include liquid amine absorption, cryogenic distillation, membrane separation, and solid adsorption, which is regarded as the most effective method for CO_2_ mitigation in the short term [2,3]. Considering its characteristics of low energy consumption and environmental friendliness [4,5], the solid adsorption method has received extensive attention from researchers. The development of an effective adsorbent with excellent CO_2_ adsorption capacity and selectivity is very important for the method. From this perspective, various adsorbents such as metal oxides [6], molecular sieves [7], nitrogen compounds [8], mesoporous silica [9], etc., have been proposed. Among them, metal–organic frameworks (MOFs), as a new type of solid adsorbent, are emphasized, owing to their large specific surface area and adjustable pore size [10], which are beneficial to CO_2_ adsorption and separation.

As a type of porous crystal, MOFs, proposed in the 1990s, are coordinated by metal ions or clusters and organic linkers [11]. Meanwhile, due to their unique properties, MOFs have been widely applied in fields including photocatalysis [12], drug delivery [13], gas separation [14], as well as chemical sensing [15]. Among them, Cu-BTC is one of the most well-known MOFs, which has been widely applied especially in CO_2_ capture since it was first prepared in 1999 [16]. Guan et al. [17] reported that the CO_2_ adsorption capacity of Cu-BTC increased with pressure but decreased with rising temperature. At 25 °C and 1.0 bar, the CO_2_ adsorption capacity reached 4.16 mmol/g. In addition, the adsorbent exhibited a high CO_2_/N_2_ (15%/85%) selectivity of 9.5, surpassing that of many conventional adsorbents. Yan et al. [18] explored the effect of solvent immersion on the CO_2_ adsorption performance of Cu-BTC. The results showed that treatment with ethanol and ammonium chloride significantly enhanced the adsorption capacity, which reached 11.6 mmol/g at 0 °C under 1 atm of pure CO_2_—a 61% improvement over the original sample.

However, the large specific surface area is not fully used for CO_2_ capture, only by physical interaction between Cu-BTC and gas molecules. Currently, the primary strategy for enhancing the CO_2_ adsorption capacity of Cu-BTC involves preparing mixed-component MOF materials. This approach mainly entails synthesizing mixed-ligand or mixed-metal MOFs or incorporating MOFs into other materials [19]. The incorporation of new metal nodes into MOFs will lead to the generation of defects as well as synergistic effects through intimate integration, which is conducive to improving the CO_2_ adsorption capacity [20]. Similarly, new metal nodes lead to an increase in the charge density of the bimetallic MOFs materials, which is also the reason for the enhanced CO_2_ adsorption [21]. Furthermore, the specific surface area, pore distribution, and other special properties of the mixed-component MOFs can be adjusted by the ratio of organic and inorganic compositions in the structure.

Hence, in this article, we proposed a mixed-component MOF prepared using the solvothermal method. The Zn-Cu-BTC/MCFs, a mixed-components Cu-BTC composite, were prepared by introducing Zn^2+^ with an electron configuration similar to Cu^2+^ and mesocellular foams (MCFs) with good hydrothermal stability to adjust its texture properties and water resistance [22]. Moreover, the composites were subject to characterizations. The single-component adsorption isotherms of CO_2_ and N_2_ were determined and fitted to the Langmuir–Freundlich model. Meanwhile, the CO_2_/N_2_ selectivity on the adsorbent was predicted based on the ideal adsorption solution theory (IAST). The Zn-Cu-BTC/MCFs exhibit excellent CO_2_ adsorption capacity and a high CO_2_/N_2_ adsorption selectivity and recycling performance.

## 2. Experimental Section

### 2.1. Chemicals and Materials

Copper nitrate trihydrate [Cu(NO_3_)_2_•3H_2_O, 99.5%] and ethanol anhydrous (EtOH, 99.7%) were provided by Beichen Fangzheng Chemical Reagent Co., Ltd. (Tianjin, China). Zinc nitrate hexahydrate [Zn(NO_3_)_2_•6H_2_O, 99%] was obtained from Beijing Bailingwei Technology Co., Ltd. (Beijing, China). Trimesic acid (H_3_BTC, 98%) was purchased from Aladdin Biochemical Technology Co., Ltd. (Shanghai, China). N,N-dimethylformamidel (DMF, 99.5%) was obtained from Sinopharm Chemical Reagent Co., Ltd. (Shanghai, China). All materials were tested directly without any treatment.

### 2.2. Preparation of the Zn-Cu-BTC/MCFs

The Cu-BTC/MCFs were prepared using the solvothermal method according to a previous study [21]. The preparation of the Cu and Zn mixed-component bimetallic MOFs composite was as follows: Firstly, 0.1 g MCFs was added into the solution in which 9.5 mmol H_3_BTC was dissolved in a 48 mL mixture (DMF:EtOH:H_2_O = 1:1:1) and stirred for 1 h (solution A). Then, 17.2 mmol metal nitrate hydrate [Zn(NO_3_)_2_•6H_2_O:Cu(NO_3_)_2_•3H_2_O = 1:1.5–1:9] was dissolved in the above 48 mL mixture to form solution B. After adding solution B to solution A and undergoing magnetic stirring for 6 h, it was transferred to a glass bottle and hydrothermally synthesized at 80 °C for 24 h. After cooling to room temperature, it was subjected to centrifugation, washing with DMF and EtOH-H_2_O mixture (*v*/*v* = 1:2), and drying to obtain a blue solid. After that, the solids were further purified by EtOH extraction. Finally, the products were activated by drying at 200 °C for 10 h under vacuum.

### 2.3. Characterization

The Fourier transform infrared spectra (FT-IR) of the adsorbents were characterized on a German Bruker TENSOR 27 Instrument (Bruker, Beijing, China). The thermodynamic behavior of the adsorbents was determined by thermogravimetric analysis (TGA) under a N_2_ atmosphere with a heating rate of 5 °C/min on a SETSYS Evolution TGA 16/18 from the France Setaram Instrument Company (Shanghai, China). The powder X-ray diffraction patterns (XRD) were recorded using the DX 2700B diffractometer (Dandong Fangyuan Instrument Co., Ltd., Dandong, China) with a scanning speed of 4 °/min. N_2_ adsorption–desorption isotherms of the adsorbents were recorded at −196 °C by an American Micromeritics ASAP 2020 automatic adsorption instrument (Micromeritics, Shanghai, China). Prior to the test, the adsorbents were degassed at 200 °C under vacuum 12 h. X-ray photoelectron spectroscopy (XPS) was carried out by an ESCALAB 250 Xi spectrometer from the American Thermo Scientific Company (Shanghai, China). The morphological characteristics were obtained using Japan’s JSM-7001F scanning electron microscopy. During the experiment, the accelerating voltage was 3 kV. The transmission electron microscopy (TEM) was obtained on a JEM-2100F instrument (JEOL, Beijing, China) operated at 200 kV.

### 2.4. Gas Adsorption and Desorption Test

The CO_2_ (99.999%) and N_2_ (99.999%) adsorption isotherms of the Zn-Cu-BTC/MCFs were collected at 35 °C and 0–7 bar on a Hiden IGA 002 Intelligent Weight Analyzer (Hiden, Beijing, China). Following a 2 h activation at 200 °C under vacuum, the adsorbents were ready for measurement. Simultaneously, the gas adsorption amounts were calculated by the mass change. Similarly, the gas desorption process was performed at 200 °C under vacuum in 2 h until no weight loss was detected for the adsorbent.

The gas adsorption capacity was determined based on the following equation:

(1) $$q_{e}=\frac{\;\left(m_{e}-m_{0}\right)\times 1000}{m_{0}\times 44}\;$$

where the *q_e_* was the equilibrium adsorption capacity (mmol/g) of the sample, and *m_e_* and *m*_0_ were the equilibrium adsorption and initial mass (mg).

## 3. Results and Discussion

### 3.1. Characterizations of Zn-Cu-BTC/MCFs

To determine the best CO_2_ adsorption amounts of the bimetallic MOF composites, different molar ratios (1:1.5–1:9) of Zn(NO_3_)_2_•6H_2_O and Cu(NO_3_)_2_•3H_2_O (the total molar amount of bimetallic was invariable) were applied for the synthesis of Zn-Cu-BTC/MCFs. As shown in Figure S1, the adsorption capacity of the materials increased with the rising pressure, indicating that they could be used in the pressure swing adsorption process. It was clear that the Zn-Cu-BTC/MCFs-1:7.5 had the highest CO_2_ adsorption capacity under 0–7 bar. Furthermore, the FT-IR spectra of the samples as displayed in Figure S2 proved that the bimetallic MOF composites were successfully synthesized. Due to the highest CO_2_ uptake, further investigations were tested on Zn-Cu-BTC/MCFs-1:7.5 which was abbreviated as Zn-Cu-BTC/MCFs. Some experiments were also performed on Cu-BTC/MCFs for comparison.

**Figure 1:**
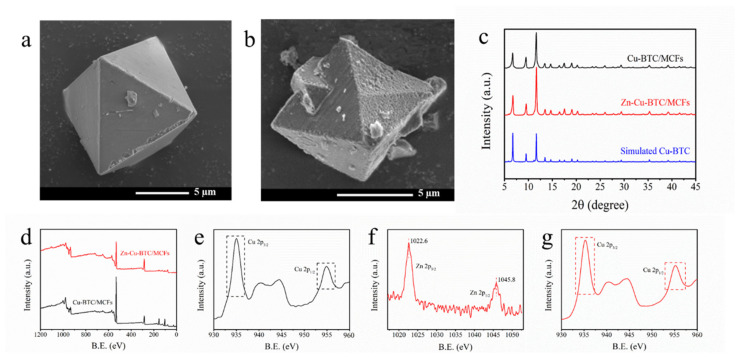
SEM images of Cu-BTC/MCFs (**a**) and Zn-Cu-BTC/MCFs (**b**); XRD patterns (**c**); XPS survey spectra (**d**); the Cu 2p spectrum of Cu-BTC/MCFs (**e**); and the Zn 2p and Cu 2p spectra of Zn-Cu-BTC/MCFs (**f**,**g**).

Figure 1a,b show the surface topography of the Cu-BTC/MCFs and Zn-Cu-BTC/MCFs, respectively. Both materials displayed typical octahedral morphologies, which were similar to Cu-BTC [23]. Simultaneously, the surfaces of the materials were covered with coral-like MCFs [24]. The grain sizes were about 10 to 15 μm. The crystal with Zn^2+^ was successfully formed and the morphology was not destroyed. The XRD patterns of the samples in Figure 1c demonstrated that the structure of the Cu-BTC/MCFs and Zn-Cu-BTC/MCFs were consistent with the simulated spectrum of Cu-BTC. The sharp diffraction peaks between 5° and 20° suggest that the as-synthesized samples possessed high purity and crystallinity [25,26]. The intensity of the diffraction peaks of Zn-Cu-BTC/MCFs were higher than that of Cu-BTC/MCFs, suggesting a larger grain size according to the Debye–Scherrer equation, which was in accordance with the SEM results. The XPS spectra of the Cu-BTC/MCFs and Zn-Cu-BTC/MCFs are shown in Figure 1d–g. The binding energies at 1022.6 and 1045.8 eV attributed to Zn 2p_3/2_ and Zn 2p_1/2_, respectively, were clearly observed from the high-resolution spectrum for the Zn-Cu-BTC/MCFs (Figure 1f) [27]. Moreover, there was no significant difference for the binding energy of Cu 2p_3/2_ and Cu 2p_1/2_ between Cu-BTC/MCFs and Zn-Cu-BTC/MCFs [Figure 1e,g]. From the results of the XRD, SEM, and XPS, it could be inferred that the second metal Zn^2+^ was simultaneously coordinated with trimesic acid as a metal ion center, forming a coordination polymer with two metal centers of copper and zinc. In addition, the surface characteristics of the Zn-Cu-BTC/MCFs were consistent with that of the Cu-BTC/MCFs, which might be related to the similar chemical characteristics of Cu^2+^ and Zn^2+^.

**Figure 2:**
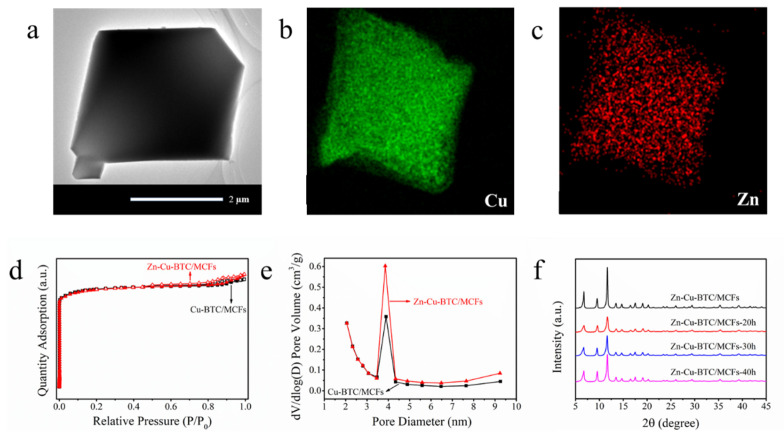
TEM image of Zn-Cu-BTC/MCFs (**a**), TEM-EDS mapping images of Cu and Zn (**b**,**c**), N_2_ adsorption/desorption isotherms (**d**), pore size distributions (**e**) of Cu-BTC/MCFs and Zn-Cu-BTC/MCFs, and XRD patterns of Zn-Cu-BTC/MCFs after exposure to water (**f**).

Figure 2a shows the TEM image of the Zn-Cu-BTC/MCFs. A typical octahedron structure could be observed, which was consistent with the SEM image. Indeed, the contrast of the light and dark areas also confirms the morphology. According to the high-resolution TEM image of the Zn-Cu-BTC/MCFs (Figure S3), the presence of Cu and Zn were observed [28,29]. The EDS element mapping for the Zn-Cu-BTC/MCFs revealed that the Cu and Zn were evenly distributed in the crystal, and the content of Cu was much higher than that of Zn [Figure 2b,c]. Due to the uniformity of Zn incorporation, it inevitably had an impact on the textural properties on the MOFs. The N_2_ adsorption and desorption isotherms of the Cu-BTC/MCFs and Zn-Cu-BTC/MCFs were obtained. It was obvious from Figure 2d that both adsorbents showed type-I isotherms [30]. A slight but clear enhancement in N_2_ adsorption capacity was observed for the Zn-Cu-BTC/MCFs compared with the Cu-BTC/MCFs. The texture properties of the Cu-BTC/MCFs and the Zn-Cu-BTC/MCFs are listed in Table 1. The BET specific surface area and total pore volume of the Cu-BTC/MCFs were 1412 m^2^/g and 0.71 cm^3^/g, respectively, which surpass that of pure Cu-BTC [31]. With the addition of Zn^2+^, the specific surface area of the Zn-Cu-BTC/MCFs also increased. Although the square planar coordination environment of the metal cations in the MOFs was not favorable for Zn^2+^ [19], the formation of a new interface between the metal center in the MOFs and the oxygen-containing functional groups in the MCFs led to an increased specific surface area. Moreover, as shown in Figure 2e and Table 1, the mesopore volume (2–10 nm) and the average pore diameter of Zn-Cu-BTC/MCFs increased compared with Cu-BTC/MCFs, which might be due to the addition of Zn^2+^ leading to greater pore accumulation. The thermal stability of the Zn-Cu-BTC/MCFs was measured using thermogravity (Figure S4c). Similarly to the pure Cu-BTC [32] and Cu-BTC/MCFs [Figure S4a], the Zn-Cu-BTC/MCFs had two weight loss steps, among which the step between 300 and 400 °C was attributed to the decomposition of the organic framework [33]. On the other hand, the water stability of the Zn-Cu-BTC/MCFs was evaluated as follows: An appropriate amount of the adsorbent was exposed to a certain amount of deionized water for several hours. After centrifugation and drying, the sample was characterized. As shown in Figure 2f, after 20, 30, and 40 h of water treatment, the XRD patterns of the Zn-Cu-BTC/MCFs exhibited no significant changes apart from a reduction in peak intensity compared with the fresh adsorbent, indicating good water stability. Notably, the crystal color changed markedly from dark blue to light blue after water exposure, which can be attributed to the coordination of water molecules (Figure S5). After vacuum activation, the original color was restored, suggesting that the adsorption process is reversible [34].

### 3.2. Gas Adsorption Isotherms

**Figure 3:**
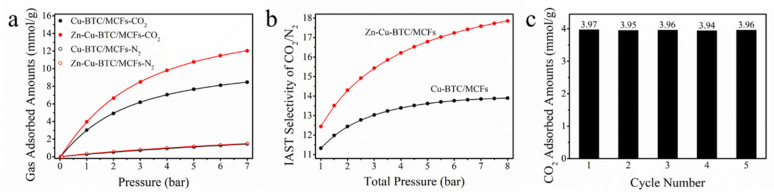
The CO_2_ and N_2_ adsorption isotherms (**a**) and IAST selectivities of CO_2_/N_2_ (**b**) of the Cu-BTC/MCFs and Zn-Cu-BTC/MCFs, and CO_2_ cyclic performance of Zn-Cu-BTC/MCFs (**c**).

The CO_2_ and N_2_ adsorption capacities of the Cu-BTC/MCFs and Zn-Cu-BTC/MCFs at 35 °C were systematically studied and are shown in Figure 3a. It could be seen that under the test conditions, the N_2_ adsorbed amounts of the adsorbents were the same, while the CO_2_ adsorption capacity of the Zn-Cu-BTC/MCFs was much higher than that of the Cu-BTC/MCFs. Among them, the Zn-Cu-BTC/MCFs exhibited a CO_2_ capacity of 3.97 mmol/g at 35 °C and 1 bar, which was an increase of 31.5% in comparison with the Cu-BTC/MCFs. The fitting of the adsorption isotherms was performed using the Langmuir–Freundlich equation [35]:

(2) $$q_{e}=\frac{q_{m}K_{L}P^{\frac{1}{n}}}{\;1+K_{L}P^{\frac{1}{n}}}$$

where *q_e_* and *q_m_* were the equilibrium and maximum adsorption capacity (mmol/g) at the pressure of *P* (bar), respectively. *K_L_* was the adsorption equilibrium constant, and *n* characterizes the difficulty of the adsorption process (with n > 1 signifying a more challenging process). The model’s accuracy was evaluated using the absolute average relative deviation (*AARD*) according to the following expression [36]:

(3) $$\mathrm{AARD}(\%)=\;\frac{1}{i}\sum_{i}\frac{\left|q_{\mathrm{simu}}-q_{\exp}\right|}{q_{\exp}}\times 100\%$$

where *i* was the number of experimental data, *q_simu_* and *q_exp_* were the simulated and experimental adsorption capacities (mmol/g), respectively. Table 2 lists the Langmuir–Freundlich parameters for the adsorption isotherms. The excellent fit of the model, as evidenced by *R^2^* ≥ 0.99 and *AARDs* ≤ 0.5%, confirms its accuracy and supports the occurrence of multilayer adsorption. A comparison reveals that the Zn^2+^ modification facilitated CO_2_ adsorption but hindered N_2_ adsorption on the Zn-Cu-BTC/MCFs. The CO_2_/N_2_ selectivity of CO_2_/N_2_ on the Cu-BTC/MCFs and Zn-Cu-BTC/MCFs (35 °C, CO_2_/N_2_ = 15:85) was further predicted based on the IAST [37]:

(4) Sads=qCO2qN2PCO2PN2

where *q_CO_*_2_ and *q_N_*_2_ were the CO_2_ and N_2_ adsorption capacities (mmol/g) of the adsorbent materials, and *P_CO_*_2_ and *P_N_*_2_ were the partial pressures (bar) in the mixture. It was found from Figure 3b that the adsorption selectivity of CO_2_/N_2_ at the Zn-Cu-BTC/MCFs was higher than that of the Cu-BTC/MCFs under the same condition. To further validate the IAST predictions, we intend to conduct additional experiments, specifically breakthrough curve measurements, in our future work. The cyclic performance of the Zn-Cu-BTC/MCFs was tested at 35 °C and 1 bar. The desorption process was conducted under an argon atmosphere at 200 °C for 2 h. As shown in Figure 3c, after five cycles of adsorption/desorption cycles, the CO_2_ adsorption capacity of the material remained basically at 3.97 mmol/g, suggesting that the Zn-Cu-BTC/MCFs had good recycling performance. Table 3 lists the comparison of the CO_2_ adsorption capacity of the Cu-BTC/MCFs and Zn-Cu-BTC/MCFs with other solid sorbents. Compared with the existing CO_2_ adsorbents, the Zn-Cu-BTC/MCFs not only had a higher CO_2_ adsorption capacity, but also had a simple preparation process; it is expected that it will become an alternative material for large-scale CO_2_ capture.

## 4. Conclusions

In summary, novel Cu and Zn mixed-component bimetallic MOF composites were prepared using a simple solvothermal method. The bimetallic MOFs had a high BET specific surface area and pore volume. The adsorption isotherms of the MOF composites were experimentally determined. With increasing pressure, the adsorbed amount of CO_2_ on the bimetallic MOF composites exhibited a corresponding increase, indicating a favorable performance under pressure swing adsorption conditions. The Zn-Cu-BTC/MCFs exhibited a CO_2_ adsorption capacity of 3.97 mmol/g at 35 °C and 1 bar, which was 31.5% higher than that of the Cu-BTC/MCFs. The Zn-Cu-BTC/MCFs also had good recyclability and better CO_2_/N_2_ adsorption selectivity according to the IAST.

## Figures and Tables

**Table 1:** The textural properties of Cu-BTC/MCFs and Zn-Cu-BTC/MCFs.

Sorbents	S_BET_ (m^2^/g)	V_total_ (cm^3^/g)	V_micro_ (cm^3^/g)	Average Pore Diameter (nm)
Cu-BTC/MCFs	1412	0.71	0.56	4.1
Zn-Cu-BTC/MCFs	1529	0.75	0.56	4.4

**Table 2:** The fitting relevant parameters of Cu-BTC/MCFs and Zn-Cu-BTC/MCFs adsorption isotherms according to the Langmuir–Freundlich equation.

Sorbents	Langmuir–Freundlich				AARD (%)
	q_m_	K_L_	n	R^2^	
Cu-BTC/MCFs-CO_2_	11.223	0.368	0.913	0.9998	0.1521
Zn-Cu-BTC/MCFs-CO_2_	16.419	0.317	0.899	0.9999	0.2099
Cu-BTC/MCFs-N_2_	6.440	0.045	1.046	0.9993	0.4939
Zn-Cu-BTC/MCFs-N_2_	6.213	0.054	1.104	0.9987	0.3301

**Table 3:** The summary of CO_2_ adsorption capacities over different solid materials.

Solid Materials	Temperature (°C)	Pressure (bar)	CO_2_ Adsorption Capacity (mmol/g)	Refs
Cu@BNNF	25	1	2.77	[38]
30PEI-HP2MGL	25	1	2.70	[39]
Ti-SBA-15	25	1	1.20	[40]
GO-MPD	25	1	0.91	[41]
MIL-53(Al)	45	1	1.14	[42]
MOF-177	25	1	1.03	[43]
MOF-505@5GO	25	1	3.94	[44]
Cu-BTC/MCFs	25	1	3.14	This work
Cu-BTC/MCFs	35	1	3.02	This work
Cu-BTC/MCFs	45	1	2.65	This work
Cu-BTC/MCFs	55	1	2.43	This work
Zn-Cu-BTC/MCFs	25	1	4.05	This work
Zn-Cu-BTC/MCFs	35	1	3.97	This work
Zn-Cu-BTC/MCFs	45	1	3.82	This work
Zn-Cu-BTC/MCFs	55	1	3.60	This work
Zn-BTC/MCFs	35	1	0.30	This work

## Data Availability

The original contributions presented in this study are included in the article/Supplementary Materials. Further inquiries can be directed to the corresponding authors.

## Supplementary Materials

The following supporting information can be downloaded at: https://www.mdpi.com/article/10.3390/nano15231777/s1, Figure S1. The CO_2_ adsorption isotherms of Zn-Cu-BTC/MCFs-x. Table S1. The fitting relevant parameters of adsorption isotherms of Zn-Cu-BTC/MCFs-x by Langmuir-Freundlich equation. Figure S2. The FT-IR spectra of Zn-Cu-BTC/MCFs-x. Figure S3. The high-resolution TEM images of Zn-Cu-BTC/MCFs. Figure S4. The TG curves of Zn-Cu-BTC/MCFs-x. Figure S5. The Comparison of whether Zn-Cu-BTC/MCFs crystals absorb water. Figure S6. The SEM images of Zn-Cu-BTC/MCFs-x. Figure S7. The XRD patterns of Zn-Cu-BTC/MCFs-x. Figure S8. The N_2_ adsorption/desorption isotherms of Zn-Cu-BTC/MCFs-x. Table S2. The textural properties of Zn-Cu-BTC/MCFs-x. Table S3. The compositions of sorbents. Figure S9. The pore size distributions of Zn-Cu-BTC/MCFs-x. Figure S10. The XRD patterns of Cu-BTC/MCFs after exposure to water.
