# Supplementary material for: A Novel Zn-Cu Bimetallic Mixed-Component MOFs Composite for Efficient CO2 Capture
Source: Nanomaterials (Basel). 2025 Nov 26;15(23):1777. doi: 10.3390/nano15231777 (PMC12693314; doi:10.3390/nano15231777)
Supplement: Supplementary file 1 [file nanomaterials-15-01777-s001.zip › nanomaterials-4000910-supplementary.pdf]

Supporting Information

A Novel Zn-Cu Bimetallic Mixed-Component MOFs Composite for Efficient CO_2 Capture

Haihong Zhao,[a, b, *] Lei Li,[c] Jiaxin Li,[a] Feiqi Yan,[a] Wenhao Wang,[a] and Mingxia Zhao[a,b,*]

[a] Department of Mining Engineering, Shanxi Institute of Technology, Yangquan 045000, China

[b] Yangquan Technology Innovation Center of Carbon Dioxide Capture, Utilization and Storage, Yangquan 045000, China

[c] State Key Laboratory of Coal Conversion, Institute of Coal Chemistry, Chinese Academy of Sciences, Taiyuan 030001, China

[*] Corresponding author: zhaohh0504@163.com; zhmx@sxit.edu.cn

Contents

Figure S1. The CO_2 adsorption isotherms of Zn-Cu-BTC/MCFs-x.

Table S1. The fitting relevant parameters of adsorption isotherms of Zn-Cu-BTC/MCFs-x by Langmuir-Freundlich equation.

Figure S2. The FT-IR spectra of Zn-Cu-BTC/MCFs-x.

Figure S3. The high-resolution TEM images of Zn-Cu-BTC/MCFs.

Figure S4. The TG curves of Zn-Cu-BTC/MCFs-x.

Figure S5. The Comparison of whether Zn-Cu-BTC/MCFs crystals absorb water.

Figure S6. The SEM images of Zn-Cu-BTC/MCFs-x.

Figure S7. The XRD patterns of Zn-Cu-BTC/MCFs-x.

Figure S8. The N_2 adsorption/desorption isotherms of Zn-Cu-BTC/MCFs-x.

Table S2. The textural properties of Zn-Cu-BTC/MCFs-x.

Table S3. The compositions of sorbents.

Figure S9. The pore size distributions of Zn-Cu-BTC/MCFs-x.

Figure S10. The XRD patterns of Cu-BTC/MCFs after exposure to water.

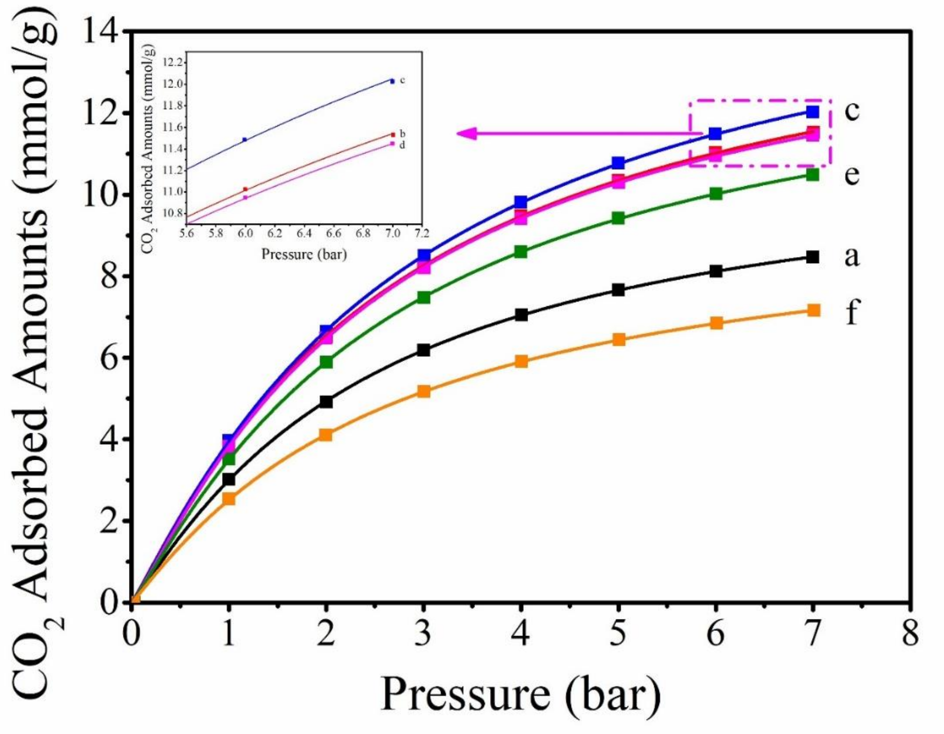

Figure S1. The CO_2 adsorption isotherms of Zn-Cu-BTC/MCFs-x.

Cu-BTC/MCFs (a), Zn-Cu-BTC/MCFs-1:9 (b), Zn-Cu-BTC/MCFs-1:7.5 (c), Zn-Cu-BTC/MCFs-1:4 (d), Zn-Cu-BTC/MCFs-1:2.5 (e) and Zn-Cu-BTC/MCFs-1:1.5 (f).

It can be seen from Figure S1 that the CO_2 adsorption capacities of all sorbents increased along with the increasing of pressure, indicating that MOFs composites were more suitable for pressure swing adsorption of CO_2. Compared with the Cu-BTC/MCFs, with the addition of Zn, the CO_2 adsorption capacities of Zn-Cu-BTC/MCFs-x increased at first then decreased. The CO_2 adsorption amount Zn-Cu-BTC/MCFs-1:7.5 was closest to that of HKUST-1 (8.20 and 8.54 mmol/g, 7 bar, 308 K). The results showed that the addition of Zn led to higher CO_2 adsorption capacity (3.97 mmol/g at 35 °C and 1 bar), an increase of 31.5% in comparison with the Cu-BTC/MCFs (3.02 mmol/g).

Table S1. The fitting relevant parameters of adsorption isotherms of Zn-Cu-BTC/MCFs-x by Langmuir-Freundlich equation.

Sorbents	Langmuir-Freundlich				AARD (%)
	q_m	K_L	n	R^2	
Zn-Cu-BTC/MCFs-1:9	15.563	0.338	0.909	0.9998	0.1725
Zn-Cu-BTC/MCFs-1:4	15.479	0.335	0.907	0.9999	0.1376
Zn-Cu-BTC/MCFs-1:2.5	14.111	0.343	0.917	0.9997	0.4343
Zn-Cu-BTC/MCFs-1:1.5	9.663	0.353	0.929	0.9998	0.1355

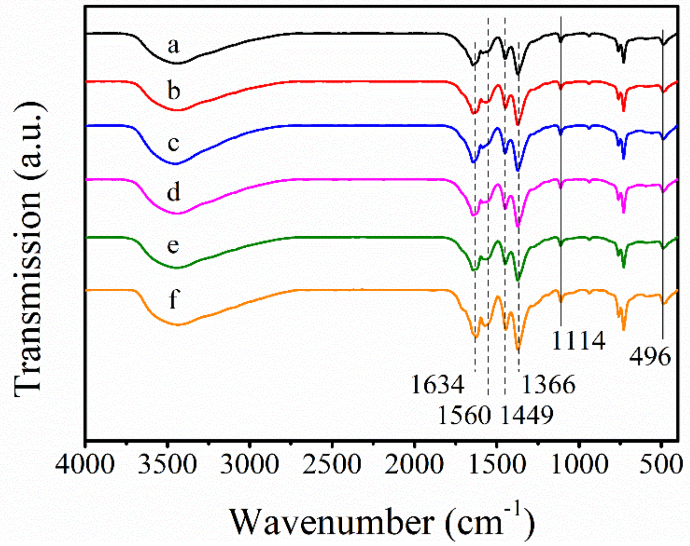

Figure S2. The FT-IR spectra of Zn-Cu-BTC/MCFs-x.

Cu-BTC/MCFs (a), Zn-Cu-BTC/MCFs-1:9 (b), Zn-Cu-BTC/MCFs-1:7.5 (c), Zn-Cu-BTC/MCFs-1:4 (d), Zn-Cu-BTC/MCFs-1:2.5 (e) and Zn-Cu-BTC/MCFs-1:1.5 (f).

The Cu-BTC/MCFs, and Zn-Cu-BTC/MCFs-x were characterized by FT-IR spectroscopy (Figure S2). It should be noted that the infrared absorption band at ca. 496 and 11144 cm^{-1} were attributed to the absorption peak of silica in MCFs. All Zn-Cu-BTC/MCFs-x composite materials exhibited characteristic peaks similar to Cu-BTC/MCFs. The symmetric and asymmetric stretching vibrations of carboxylate

groups in H_3BTC appeared at 1634/1560 cm^{-1} and 1449/1366 cm^{-1}, respectively. The results revealed that the materials were successfully synthesized.

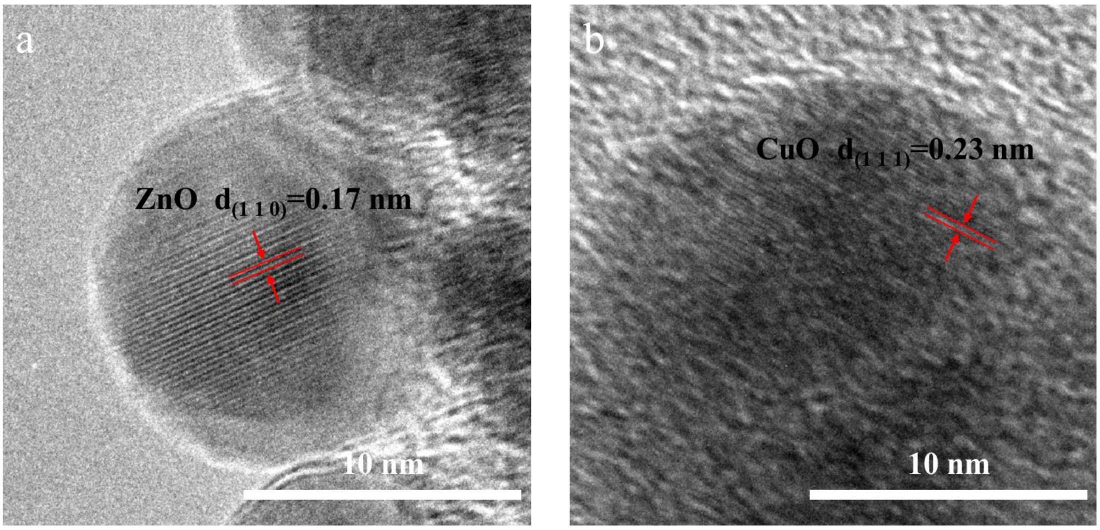

Figure S3. The high-resolution TEM images of Zn-Cu-BTC/MCFs.

(ZnO: PDF#36-1451, and CuO: PDF#48-1548)

As shown in Figure S3, the high-resolution TEM images of Zn-Cu-BTC/MCFs was performed and the d-spacing of ZnO and CuO were measured. (ZnO $d_{(1\ 1\ 0)}$ = 0.17 nm: PDF#36-1451, and CuO $d_{(1\ 1\ 1)}$ = 0.23nm: PDF#48-1548) It had proved the high degree of crystallization of MOFs.

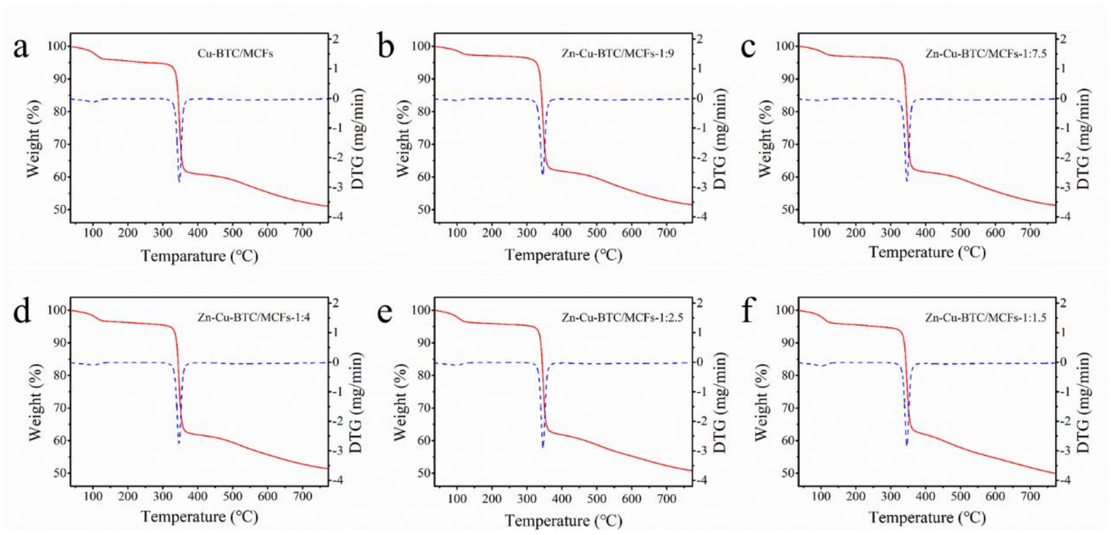

Figure S4. The TG curves of Zn-Cu-BTC/MCFs-x.

The TG curves of the Zn-Cu-BTC/MCFs-x were demonstrated in Figure S4. It can be seen that the TG curves of all samples were completely in agreement with those of Cu-BTC/MCFs, which exhibited two weight loss steps. The first weight loss step below 250 °C could be attributed to the removal of physical adsorption of water and small organic molecules in the frameworks. The second weight loss step might due to the decomposition of the organic framework of the sorbents occurred in the temperature range of 300–400 °C. Given that the MCFs did not decompose before 550 °C, the amounts of MCFs for the Zn-Cu-BTC/MCFs-x could be estimated according to the weight loss ratio of the sorbents, which was 11.47%.

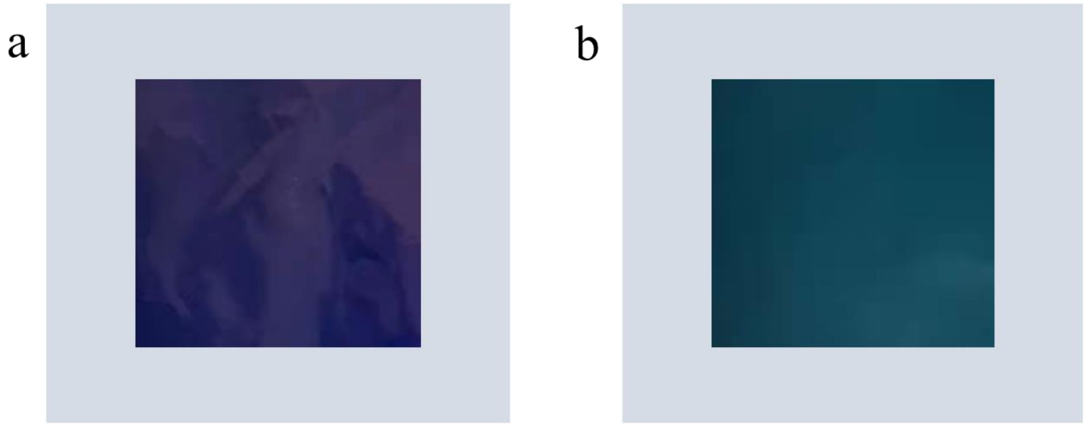

Figure S5. The Comparison of whether Zn-Cu-BTC/MCFs crystals absorb water. fresh sample (a), and samples adsorbed water (b).

It was worth noting that the color of the crystals changed significantly from dark blue to light blue due to the coordination of water molecules after water treatment [Figure S5]. After activation under vacuum, the color of the crystal could be restored, indicating that it was a reversible adsorption process.

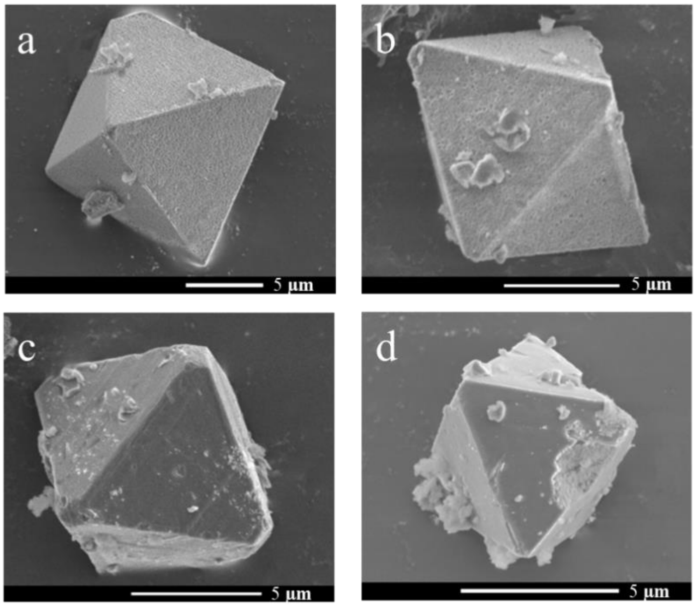

Figure S6. The SEM images of Zn-Cu-BTC/MCFs-x.

Zn-Cu-BTC/MCFs-1:9 (a), Zn-Cu-BTC/MCFs-1:4 (b), Zn-Cu-BTC/MCFs-1:2.5 (c) and Zn-Cu-BTC/MCFs-1:1.5 (d).

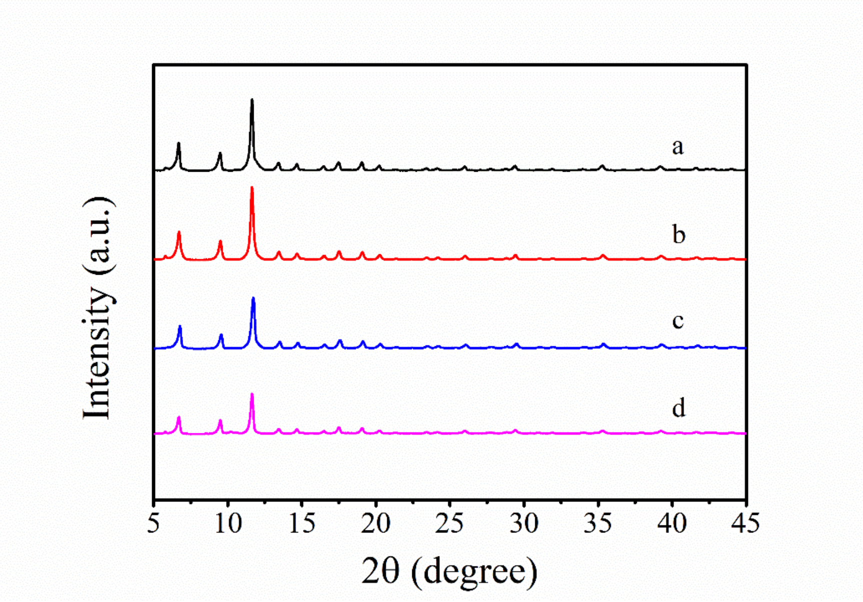

Figure S7. The XRD patterns of Zn-Cu-BTC/MCFs-x.

Zn-Cu-BTC/MCFs-1:9 (a), Zn-Cu-BTC/MCFs-1:4 (b), Zn-Cu-BTC/MCFs-1:2.5 (c) and Zn-Cu-BTC/MCFs-1:1.5 (d).

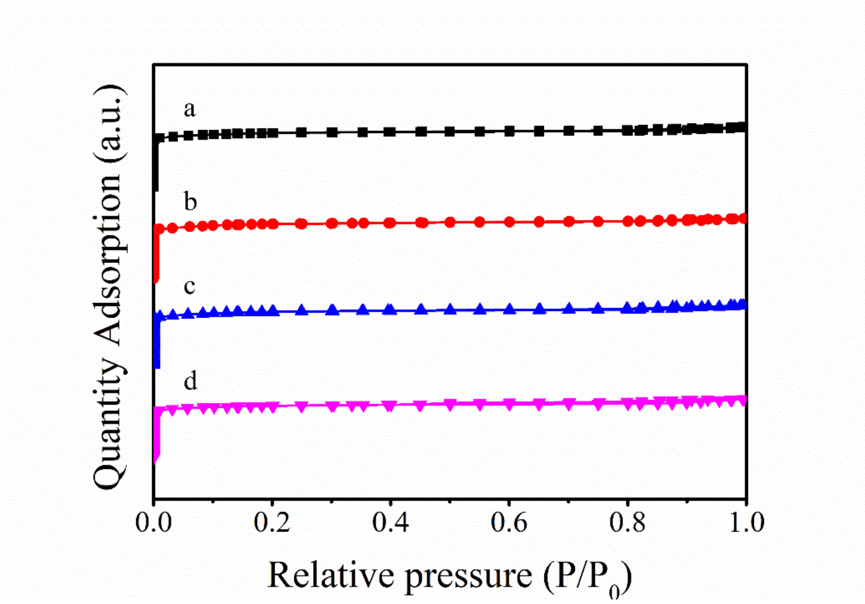

Figure S8. The N2 adsorption/desorption isotherms of Zn-Cu-BTC/MCFs-x.

Zn-Cu-BTC/MCFs-1:9 (a), Zn-Cu-BTC/MCFs-1:4 (b), Zn-Cu-BTC/MCFs-1:2.5 (c) and Zn-Cu-BTC/MCFs-1:1.5 (d).

Table S2. The textural properties of Zn-Cu-BTC/MCFs-x.

Sorbents	S_{BET} (m^2/g)	V_{total} (cm^3/g)	V_{micro} (cm^3/g)	Average pore diameter (nm)
Zn-Cu_BTC/MCFs-1:9	1464	0.75	0.57	4.4
Zn-Cu-BTC/MCFs-1:4	1480	0.75	0.57	4.3
Zn-Cu-BTC/MCFs-1:2.5	1447	0.74	0.56	4.4
Zn-Cu-BTC/MCFs-1:1.5	1418	0.74	0.56	4.2

Table S3. The compositions of sorbents

Sorbents	Compositions (%)		Zn/Cu atomic ratio
	Zn	Cu	
Zn-Cu BTC/MCFs-1:9	10.3	89.7	1:8.71
Zn-Cu-BTC/MCFs-1:4	19.5	80.5	1:4.13
Zn-Cu-BTC/MCFs-1:2.5	28.6	71.4	1:2.50
Zn-Cu-BTC/MCFs-1:1.5	40.7	59.3	1:1.48

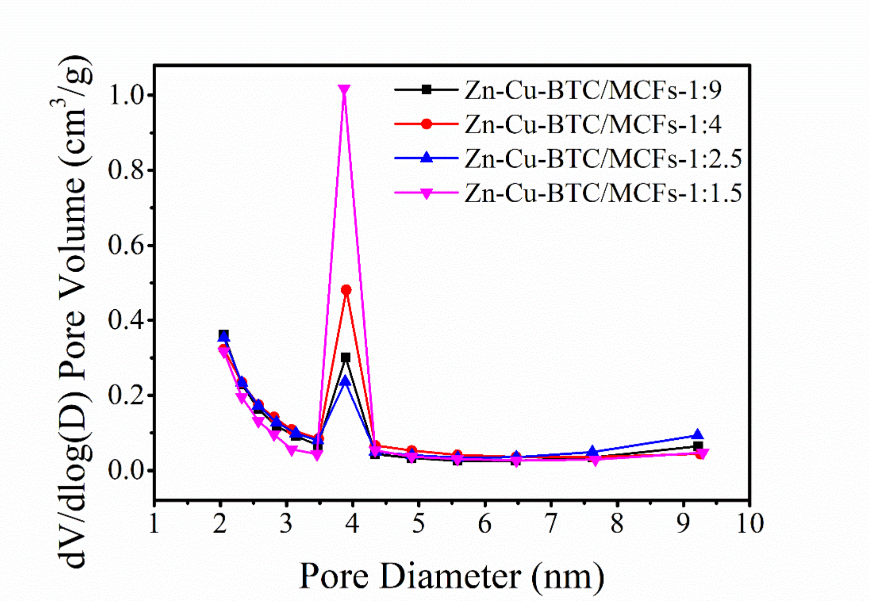

Figure S9. The pore size distributions of Zn-Cu-BTC/MCFs-x.

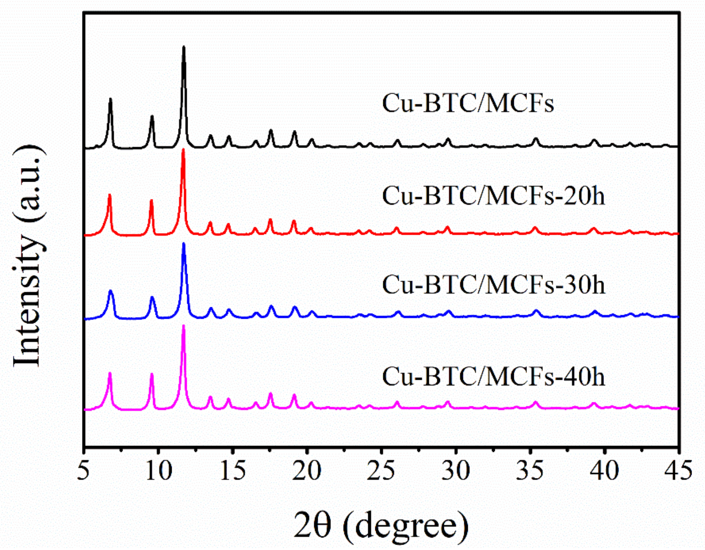

Figure S10. The XRD patterns of Cu-BTC/MCFs after exposure to water.
